# Giant Echinococcosis of the Liver with Suppuration: A Case Report and Review of the Literature

**DOI:** 10.3390/medicina59061070

**Published:** 2023-06-02

**Authors:** Christoforos S. Kosmidis, Konstantinos Papadopoulos, Chrysi Maria Mystakidou, Christina Sevva, Charilaos Koulouris, Nikolaos Varsamis, Stylianos Mantalovas, Vasileios Lagopoulos, Vasiliki Magra, Vasiliki Theodorou, Styliani Ouzouni, Nikolaos Iason Katsios, Paraskevi Axi, Konstantinos Sapalidis, Isaak Kesisoglou

**Affiliations:** 1 European Interbalkan Medical Center, 10 Asklipiou Street, 55535 Pylaia, Greece; dr.ckosmidis@gmail.com (C.S.K.); charilaoskoulouris@gmail.com (C.K.); nikvar83@gmail.com (N.V.); 2 3rd Surgical Department, University General Hospital of Thessaloniki “AHEPA”, School of Medicine, Faculty of Health Sciences, Aristotle University of Thessaloniki, 1st St. Kiriakidi Street, 54621 Thessaloniki, Greece; christina.sevva@gmail.com (C.S.); steliosmantalobas@yahoo.gr (S.M.); vaslag@gmail.com (V.L.); valia.magra@gmail.com (V.M.); axi.paraskevi@gmail.com (P.A.); sapalidiskonstantinos@gmail.com (K.S.); ikesis@hotmail.com (I.K.); 3 Medical School, Faculty of Health Sciences, Aristotle University of Thessaloniki, 54124 Thessaloniki, Greece; chryssa2000@gmail.com (C.M.M.); baswtheodorou@hotmail.com (V.T.); stellauzouni@hotmail.gr (S.O.); 4 Medical School, Faculty of Health Sciences, University of Ioannina, 45110 Ioannina, Greece; nickkatsios@hotmail.gr

**Keywords:** cystic echinococcosis, *Echinococcus*, giant-suppurated hydatid cyst, hydatid disease, *Echinococcus granulosus*

## Abstract

*Purpose:* Cystic echinococcosis (CE) is a common, complex parasitic disease that constitutes a major public health concern. CE demonstrates high endemicity in areas where dogs are used for herding or where animal husbandry practices involve close contact with livestock. It can clinically manifest with a variety of signs and symptoms, such as cholangitis, jaundice, pancreatitis, external biliary fistula, inferior vena cava obstruction, portal hypertension, and superinfection. The latter can notably be related to suppuration, either by rupture or bacteremia. The aim of this study is to report our 76-year-old patient who presented with a primarily infected giant-suppurated hydatid cyst of the liver and its surgical management. *Methods:* In this case, the diagnosis was based primarily on clinical presentation, computed tomography (CT) scan, and magnetic resonance imaging (MRI) of the patient’s abdomen. The surgical procedure of choice was the partial retaining of the pericystic membrane and drainage of the cystic contents (partial pericystectomy). *Results:* The surgical management and meticulous long-term follow-up of our patient produced a positive outcome without any post-operative complications.

## 1. Introduction

Echinococcal cysts are fluid-filled sacs caused by the larvae of the tapeworm *Echinococcus granulosus*. These cysts can develop in various organs of the body, but the most common sites are the liver and lungs [1]. There are many complications resulting from hydatid disease, which affects almost 40% of cases [1], including hepatic hydatid cyst rupture, superinfection, and novel cyst formation in adjacent organs, such as the portal vein, biliary tree, stomach, and right diaphragm [2,3]. Suppuration occurs when the sterile hydatid fluid within the cyst becomes infected with bacteria, which can occur when intact cysts are invaded by bacteria either through small peripheral communications with the biliary system or, rarely, through hematogenous spread, which is called primary infection. Secondary bacterial infection of hydatid cysts refers to cases where the former have not been properly treated (rupture) or evacuated, and an obvious source of infection can be identified, such as a sinus tract communicating with the biliary tree, peritoneal cavity, bronchi, alimentary tract, or skin (bacteremia) [4,5]. The most common causative organism is *Staphylococcus aureus*, followed by *Escherichia coli*, *Pseudomonas aeruginosa*, and *Klebsiella pneumoniae*, *enterococcus* Spp. [4,5,6].

The formation of suppurated cysts is a rare but serious complication of echinococcal cysts. Suppurative echinococcal cysts are often more difficult to diagnose than uncomplicated cysts because they may present with nonspecific symptoms and imaging findings. In this case report, we present a patient who visited the third surgical clinic at the AHEPA hospital center in Thessaloniki with a large suppurative hepatic echinococcal cyst and its management with the partial pericystectomy technique.

## 2. Case Presentation

**Figure 1:**
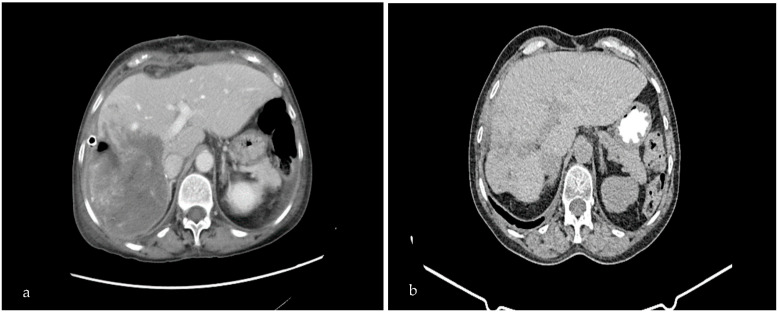
Pre-operative (**a**) and post-operative (**b**) abdominal CT scan. Pre-operative CT demonstrates the suppurated echinococcal cyst in segments V, VI, VII, and VIII of the right liver lobe, with d: 17 × 14 × 11 cm. Eight-month post-operative CT scan shows deterioration of the remaining cavity of the suppurated hydatid cyst.

We proceeded to an extensive review of a 76-year-old patient who was admitted to our hospital center, presenting with right upper quadrant pain, which had initially appeared 3 months before, accompanied by fever (38–39 °C) and weight loss of 30 kgs in a period of 9 months and a sense of fullness. The patient complained of weakness, which hindered her daily activities. At the time of presentation, there was no sign of jaundice. Consequently, she underwent a CT scan and MRI, which demonstrated an oval transversal lesion measuring 17 × 14 × 11 cm (hydatid cyst) (Figure 1a) and occupying the right hepatic lobe. Furthermore, MRI imaging illustrated the dilation of the bile duct towards the IV and VI sectors of the liver. The MRI showed no pulmonary parenchyma involvement and a lack of enlarged lymph nodes. Laboratory tests revealed anemia (Hb: 10 mg/dL) and an increased white blood cell count (WBC: 16,000 K/μL), of which 87% were neutrophils. Elisa testing for Echinococcus was positive. Upon further inquiry, the patient revealed that she lived in a rural area and she was in close contact with sheep and dogs. Thus, according to the former data and based on the WHO criteria (daughter cyst in the solid matrix, Ce3b, WHO stage), we opted for a partial pericystectomy (removal of the endocyst and partially of the pericyst), which exhibits low morbidity and mortality rates when performed by an experienced surgeon.

The patient was started on intravenous antibiotics for her current infection, which included ciprofloxacin and metronidazole, for the duration of her hospital stay and prior to her operation.

**Figure 2:**
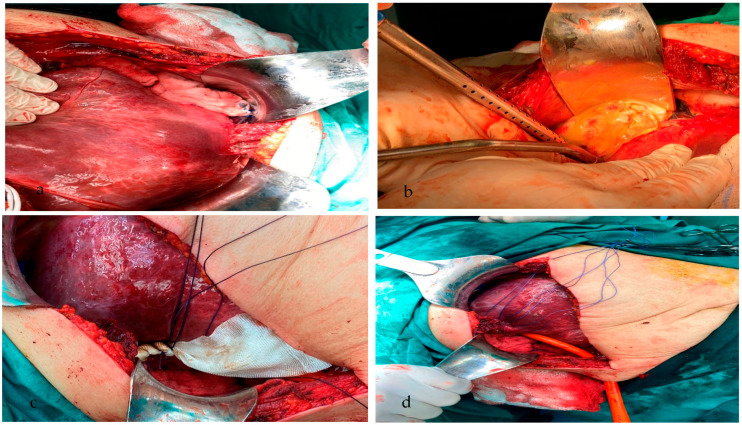
Intra-operative figures. The suppurated hydatid cyst covered with pads soaked with hypertonic saline, 15% (**a**), drainage of the cystic content (**b**), placement of hemostatic sutures and between the bile ducts and the cystic wall in order to prevent leakage of bile or ascetic fluid (**c**), and placement of the Pezzer tube in the rest of the cavity after partial pericystectomy (**d**).

Under general anesthesia, the patient underwent surgery. Through a right Kocher incision, a huge suppurative lesion (hydatid cyst, 17 × 14 × 11 cm) was revealed, occupying the right hepatic lobe (V, VI, VII, and part of the VIII sector), confirming the findings of the screening she had previously undergone. The surrounding of the cyst was covered with pads soaked with hypertonic saline, 15% (Figure 2a). Consequently, the surgical procedure included partial pericystectomy and drainage of the cystic content (1800 mL), part of which was sent for cytological examination (Figure 2b). Afterward, the peritoneal cavity was rinsed with hypertonic saline, 15%. We continued by placing hemostatic sutures at certain sites, in which there were minimal ruptures between the bile ducts and the cystic wall. The entirety of the residual cystic wall was coated with Bioglue in order to prevent leakage of bile or ascetic fluid (Figure 2c). The surgical wound was closed with Vicryl 0-1 sutures, and a Pezzer tube no. 8 was inserted (Figure 2d). We also inserted 2 Robdrain drainage tubes no. 30, one sub-diaphragmatically and one into the foramen of Winslow. The patient tolerated the procedure well, without any post-operative complications arising. Cystic fluid culture revealed the presence of *Enterococcus* spp. and confirmed our diagnosis. The patient was discharged on the 5th post-operative day and was prescribed specific anthelminthic and antibiotic medication.

Furthermore, the patient underwent post-operative treatment with 6 cycles of albendazole. Albendazole chemotherapy was found to be the primary pharmacological treatment to consider in the medical management of cystic echinococcosis and is, in general, used for reducing cysts, decreasing infection, and avoiding relapses. A follow-up CT scan of the patient 8 months post-operatively revealed deterioration of the remaining cavity of the suppurated hydatid cyst (Figure 1b).

## 3. Discussion

Cystic hydatidosis has created a global health concern and is still a major healthcare problem in Greece. The two main species of Echinococcus are *Echinococcus granulosus* (*E. granulosus*), causing cystic echinococcosis (CE), and *Echinococcus multilocularis*, causing alveolar echinococcosis (AE). Areas of the world that exhibit a high rate of infection with CE include Central and North Europe, Asia, and North America [7,8,9], whereas many new CE cases are diagnosed annually in South America [1,10,11], such as in Brazil, Argentina, and Uruguay [12]. Furthermore, there is evidence of new endemic areas, such as Belgium, Poland, and the Netherlands [10], where people live in close proximity to domestic or wild canids [13,14]. AE is found in the northern hemisphere, particularly in the Arctic and sub-Arctic regions of Europe, Asia [15], and North America [11]. In Europe, its prevalence is high, and AE cases have doubled, especially in some areas such as France, Switzerland, Germany, and Austria [7,11,16]. Additionally, hydatid disease causes a noteworthy economic impact on these endemic areas due to the need for preventative measures, research programs, and new medications, as well as the diagnosis and management of chronic clinical complications [7,10,13,14,17].

Echinococcosis is most commonly caused by *Echinococcus granulosus*, a parasitic organism that relies on its host for survival. The cysts formed by the parasite are thought to evade the host immune system by producing immunomodulatory molecules, such as cystatin and a protein called antigen B [17]. The cysts can also cause damage to the host tissue by compressing surrounding structures [4,11], inducing fibrosis, and promoting the formation of new blood vessels (angiogenesis). In addition to the aforementioned mechanisms, cystic echinococcosis also involves nutrient acquisition and metabolic adaptations by the parasite.

Thus, as we mentioned, suppurative echinococcal cysts can occur due to a primary or secondary cause [1,8]. Once the hydatid fluid is infected, an inflammatory response is initiated. This leads to the formation of a fibrous capsule around the cyst, which can become thickened and calcified over time [8], except for cases of pulmonary echinococcosis. The purulent material within the cyst can cause significant damage to surrounding tissues, including the liver or lung parenchyma. It can also lead to the development of an abscess [1,5]. In some cases, the infection can spread to other organs or into the bloodstream, leading to sepsis, a life-threatening condition.

Hydatid disease manifests with a wide range of signs and symptoms, depending on the location, size, and number of cysts in the body. Many people may be asymptomatic for years, while others may experience severe and potentially life-threatening complications [8,18]. Signs and symptoms may include abdominal pain and discomfort, nausea and vomiting, loss of appetite and weight loss, fatigue, weakness, and jaundice [3,5,9,11,18]. Large echinococcal cysts may cause obstruction of the bile duct, leading to swelling and inflammation of the liver, with consequent obstructive jaundice, cholangitis, or external biliary fistula [15,16,19]. Obstruction of the portal vein can lead to portal hypertension or even Budd Chiari syndrome due to the displacement of the inferior vena cava and hepatic veins [1,8,19,20].

Ruptured cysts can cause severe pain, anaphylactic shock, or even death. Patients with a known history of echinococcal cysts who present with fever, chills, abdominal pain, or general malaise should raise suspicion of a possible suppuration [8,16]. Furthermore, rupture of the suppurated hydatic cyst can have life-threatening consequences. Leakage of the purulent cystic content into the biliary tract or the peritoneal/pleural cavity can lead to secondary infections, abscess formation, or even anaphylaxis and septic shock [5,20]. On the other hand, bacteremia can arise due to hematogenous dissemination from numerous other anatomical regions [2,5,6,12,21,22]. Risk factors for bacteremia include cirrhosis, cancer, and diabetes mellitus [4].

Imaging tests are essential for the diagnosis of CE and may include ultrasonography, a CT scan, or MRI in order to visualize the cyst and assess its location, size, and morphology [3,5,22]. CT is the gold standard approach. Echinococcal cysts usually appear as well-defined round or oval cystic lesions with thick, smooth walls and a homogeneous low attenuation content. The cysts may also contain internal septations or calcifications, which are more commonly seen in older cysts [18]. In cases of suppurative echinococcal cysts, CT scans may reveal thick irregular walls, heterogeneous attenuation, and/or the presence of gas within the cystic content. CT scans may also help differentiate between cystic echinococcosis and other hepatic lesions, such as hepatic abscess, cystadenoma, or cystadenocarcinoma [23]. Additionally, serological tests with high sensitivity and specificity in detecting antibodies against the parasite (an enzyme-linked immunosorbent assay (ELISA)) [12,15,18,19] and immunoblotting [16,19] can aid in confirming the diagnosis.

Laboratory tests may be useful in the diagnosis and management of suppurative echinococcosis, but the results may not be specific or sensitive to this condition. A patient during a complete blood count may exhibit leukocytosis and/or eosinophilia [5], yet these findings have low specificity and may also occur in other inflammatory conditions. Hence, we took into consideration liver function tests involving the levels of liver enzymes, such as the alanine aminotransferase, aspartate aminotransferase, alkaline phosphatase, and gamma-glutamyl transpeptidase [12,19]. These enzymes can be elevated due to hepatocellular damage or biliary obstruction caused by the cysts. However, these findings are also not specific to suppurative echinococcosis. Increased C-reactive protein (CRP) and the erythrocyte sedimentation rate (ESR) in patients with known cystic echinococcosis are indicative of an intense inflammatory response and should raise concern over possible sepsis [24,25].

A consensus regarding the optimal treatment for cystic echinococcosis has not yet been reached. A number of surgical and non-surgical options have been applied, although surgery is still the main therapeutic approach when possible, as it can immediately lead to a complete cure [11,22]. The size of the cyst and its location and accessibility, as well as the presence of complications, should all be taken into consideration when selecting the appropriate surgical technique, either with open, laparoscopic, or robotic procedures. Thus, numerous factors can lead a general surgeon to follow an open procedure, such as recurrent, multiple, multilocular, and calcified cysts and their communication with the biliary tract and major vessels (the portal vein, inferior vena cava, etc.). The laparoscopic technique is preferred for unilocular, superficial, and accessible hydatid cysts with a size <5 cm, while robotic procedures are selected by many surgeons for unilocular cysts >5 cm [26].

The optimal techniques have been surgical excision via a radical or conservative approach [3,27]. Nowadays, the implementation of chemotherapy in the therapeutic regimen, using albendazole, an anthelmintic medication, offers the advantage of preventing the proliferation of the worm and lowering the risk of disease recurrence due to either inadequate cyst removal or a previously undetected cyst. Avoiding any leaks of the cystic contents in the peritoneal cavity and the cautious removal of the parasite are the main objectives of the surgical management of primary hydatid cysts [3,18,27].

Radical approaches involve total pericystectomy, partial hepatectomy, or lobectomy, which present a lower risk of recurrence and post-operative complications but higher morbidity and mortality rates [15,16,18,27], most commonly due to the lack of the surgeon’s experience. Conservative procedures include partial pericystectomy with the drainage of the cyst, during which the endocyst and part of the pericyst are removed. The latter is the technique our surgical team followed. Other conservative methods include simple tube drainage, marsupialization, the capitonnage technique with or without an omentoplasty, and the management of the remaining cystic cavity [3,18]. Partial pericystectomy is most commonly used due to the cyst’s large size, the presence of inflammatory adhesions, and the close relation between the echinococcal cyst and anatomical regions such as the biliary tree, portal vein, or inferior vena cava [11,26,27]. The aforementioned factors can lead to unsafe total cystectomy and increase the possibility of iatrogenic intra-operative complications [26,28].

Conservative techniques are accompanied by low morbidity and mortality rates compared to radical ones, especially when performed by an experienced surgeon [16]. However, they may have higher post-operative relapses due to the inadequate removal of the cyst. The PAIR (percutaneous aspiration, injection, re-aspiration) technique is a third treatment option that can be effective by itself, yet it is usually combined with albendazole for greater clinical and parasitological efficacy. PAIR is a method of choice for patients unwilling to undergo surgery and for cysts measuring >5 cm, except those with inactive or calcified cystic lesions, according to the Gharbi and WHO classifications [1,15,18,19].

Nevertheless, despite the above operative techniques, another medical approach that can lead to a complete cure or significant decrease in the size of the cyst is chemotherapy with benzimidazole compounds. Chemotherapy is indispensable due to high recurrence rates and a number of possible complications following surgical techniques. Based on the WHO suggestions, medical treatment should be initiated 4–30 days prior to the surgical approach and continued for at least 1 month thereafter for albendazole. The dose of albendazole is 10–15 mg/kg per day, and the treatment duration is 3–6 months [16,19,22]. Albendazole reduces the recurrence rate of hydatid disease and cyst fluid spillage complications after surgery. Side effects of albendazole involve headache, nausea, hair loss, and hepatotoxicity. (1). Contraindications of the above treatment include pregnancy, chronic hepatic and renal diseases, and bone marrow depression [7,16,18,19].

The main challenge of cystic echinococcosis remains treatment failure. Despite the WHO recommendations, treatment follow-up guidelines are still controversial. The dose and duration of medical treatment remain undefined. It is undeniable that the post-surgery follow-up of CE patients is necessary in order to avoid possible relapse and detect newly forming cysts in their initial stage or identify previously undetected but still viable cysts. This can be achieved by detecting the persistence of anti-E granulosus antibodies using serology tests, including latex agglutination, passive hemagglutination, immunoelectrophoresis, and specific IgE, IgM, and IgG enzyme-linked immunosorbent assays [17]. It is generally recommended to perform these tests at 3 months, 6 months, and 12 months post-operatively and then annually for up to 3 years [11,26]. Furthermore, other important tools that are used for proper follow-up involve imaging tests such as CT scans and MRIs. The frequency of imaging tests depends on the size and location of the cyst, but it is generally recommended to perform them at 6 months and 12 months post-operatively and then annually for up to 5 years. In our case, the Pezzer tube was removed after 6 months, and the patient underwent an abdominal CT scan, which demonstrated no signs of post-operative relapse or newly formed cysts.

## 4. Conclusions

The treatment of suppurated echinococcal cysts is a complex matter. The type of treatment depends on the activity of the disease, symptoms, clinical manifestation, and, of course, the performance status of the patient. Imaging tests, and specifically CT scans, constitute the gold standard approach, yet, laboratory tests can aid in instances where imaging results are inconclusive. According to the initial treatment of the patient’s disease, we should adhere to specific monitoring and possible antiparasitic protocols, following the WHO guidelines. Echinococcosis remains a challenge for the surgical team, especially in the Greek data, where the prevalence of the disease is higher. Its successful treatment requires the combination of the expertise of the surgical team in the management of hepatobiliary cases, excellent knowledge of the guidelines, and experience with echinococcosis.

## Data Availability

Not applicable.
